# TCR and CD28 Concomitant Stimulation Elicits a Distinctive Calcium Response in Naive T Cells

**DOI:** 10.3389/fimmu.2018.02864

**Published:** 2018-12-04

**Authors:** Fan Xia, Cheng-Rui Qian, Zhou Xun, Yannick Hamon, Anne-Marie Sartre, Anthony Formisano, Sébastien Mailfert, Marie-Claire Phelipot, Cyrille Billaudeau, Sébastien Jaeger, Jacques A. Nunès, Xiao-Jun Guo, Hai-Tao He

**Affiliations:** ^1^Aix Marseille University, CNRS, INSERM, CIML, Marseille, France; ^2^School of Economics, Jiangxi University of Finance and Economics, Nanchang, China; ^3^Aix Marseille University, AMSE and GREQAM, Marseille, France; ^4^Aix Marseille University, CNRS, INSERM, Institut Paoli-Calmettes, CRCM, Marseille, France; ^5^Equipe Labellisée Fondation pour la Recherche Médicale, Centre de Recherche en Cancérologie de Marseille, Immunology and Cancer, Marseille, France

**Keywords:** T cell activation, naive T cells, co-stimulation, CD28, TCR–T cell receptor, calcium signaling

## Abstract

T cell activation is initiated upon ligand engagement of the T cell receptor (TCR) and costimulatory receptors. The CD28 molecule acts as a major costimulatory receptor in promoting full activation of naive T cells. However, despite extensive studies, why naive T cell activation requires concurrent stimulation of both the TCR and costimulatory receptors remains poorly understood. Here, we explore this issue by analyzing calcium response as a key early signaling event to elicit T cell activation. Experiments using mouse naive CD4^+^ T cells showed that engagement of the TCR or CD28 with the respective cognate ligand was able to trigger a rise in fluctuating calcium mobilization levels, as shown by the frequency and average response magnitude of the reacting cells compared with basal levels occurred in unstimulated cells. The engagement of both TCR and CD28 enabled a further increase of these two metrics. However, such increases did not sufficiently explain the importance of the CD28 pathways to the functionally relevant calcium responses in T cell activation. Through the autocorrelation analysis of calcium time series data, we found that combined but not separate TCR and CD28 stimulation significantly prolonged the average decay time (τ) of the calcium signal amplitudes determined with the autocorrelation function, compared with its value in unstimulated cells. This increasement of decay time (τ) uniquely characterizes the fluctuating calcium response triggered by concurrent stimulation of TCR and CD28, as it could not be achieved with either stronger TCR stimuli or by co-engaging both TCR and LFA-1, and likely represents an important feature of competent early signaling to provoke efficient T cell activation. Our work has thus provided new insights into the interplay between the TCR and CD28 early signaling pathways critical to trigger naive T cell activation.

## Introduction

T cell activation involves two types of signal transmitted by surface receptors upon engagement with their respective ligands, which are present on antigen-presenting cells (APCs) (1). The first signal is induced via the T cell receptor (TCR) upon binding to antigenic peptide–major histocompatibility complex (pMHC); the second signal is induced via costimulatory receptors, the prototype of which is CD28, binding to either B7-1 (also known as CD80) or B7-2 (also known as CD86). CD28 is the only B7 receptor constitutively expressed on naive T cells. Costimulation through CD28 is critically required for naive T cells to achieve clonal expansion, cytokine production and for protection against apoptosis and anergy (2, 3). However, despite many years of study, the contributions from and relationship between the TCR and CD28-mediated pathways remains to be elucidated. In particular, a long-debated question in the field is: does CD28 mainly work to increase the activation of TCR-induced signaling pathways, or does it allow triggering of some unique intracellular events that are absent when only the TCR is stimulated? In this context, it has been shown that the CD28-mediated signaling—that is, signal 2—can decrease the threshold of sensitivity of TCR signaling—that is, signal 1—upon binding to pMHC, but this seems due to the modulation of TCR signaling events downstream of receptor-proximal steps (4). Moreover, CD28 stimulation in general has no effect on the “down-regulation” of TCRs upon binding to antigen (5), a surrogate marker for TCR engagement (6). In addition, engagement of CD28 at surface of T cells appears not modifying formation of the immunological synapse (IS) (7), but could impact TCR *in situ* location at T cell/APC contacts (8–11). Nevertheless, increase of signal-1-pathway activation by signal 2 does not easily explain how the presence of signal 2 prevents naive T cells from the anergy that occurs following activation of signal 1 alone. It is therefore considered that CD28 contributes both quantitatively and qualitatively to the signaling pathways driving T cell activation (2). On the other hand, recent studies conducted in antigen-experienced T cells suggested that TCR engagement can facilitate CD28–B7 interactions (12, 13) and consequently favors the costimulatory signal initiation. Sanchez-Lockhart et al. (12, 13) found that TCR stimulation, in previously activated T cells, could enhance the avidity of CD28–B7 binding via a mechanism involving a possible rotation of the ligand binding interface of the extracellular domain of CD28 homodimer. In the context of the regulation of CD28 ligand avidity, Bromley et al. (7) showed that the interactions between CD28 on naive T cells and B7 molecules on APCs are extremely weak. It was also proposed that TCR triggering produces a microenvironment at the immunological synapse that favors the interactions of potent secondary signaling molecules, such as CD28.

In many of the previous studies, the CD28-mediated (and to some degree also the TCR-mediated) signaling pathways were investigated in T cell lines or antigen-experienced T cells, but this was rarely performed in naive T cells. However, it is not clear to what extends the information obtained from these studies can apply to the activation of naive T cells. Here, we investigated the interactions between TCR- and CD28-mediated early signaling pathways upon engagement with their respective ligands and evaluated their contribution to T cell activation in mouse naive CD4^+^ T cells. By analyzing the autocorrelation function of the signal, we showed for the first time that concurrent TCR and CD28 stimulation, but not the individual stimuli, significantly prolonged the average decay time (τ) of calcium signal amplitudes, as compared with its value found in unstimulated cells. This unique costimulatory function likely contributes to TCR- and CD28-mediated signaling responses leading to efficient T cell activation. Thus, we showed calcium fluxes as a potentially important step through which TCR and CD28 early signaling pathways interact and cooperate each other for the effective initiation of naive T cell activation.

## Materials and Methods

### Mice and Ethics Statement

This study has been approved by the following Animal Care and Use Committee: Departmental Direction of Veterinary Services of Bouches du Rhône (Direction Départementale des Services Vétérinaires des Bouches du Rhône), and the approval number is F13-055-10. The study was carried out in strict accordance with the recommendations in the Guide for the Care and Use of Laboratory Animals the French Ministry of Agriculture and of the European Union. Mice were housed under specific pathogen-free conditions and handled in accordance with French and European directives. Animals were housed in the CIML animal facilities accredited by the French Ministry of Agriculture to perform experiments on alive mice. All efforts were made to minimize animal suffering. 3A9 TCR transgenic (TCRtg) mice (14) maintained on a CBA/J background were bred onto a CBA/J x C3H/HeN before used in the experiments (15). C3H/HeN mice were purchased from Janvier Labs SAS (Le Genest St Isle, France). Sex and age of mice were varying according to the experiment performed.

### Reagents and Antibodies

4-amino-5-(4-chlorophenyl)-7-(dimethylethyl) pyrazolo [3,4-d] pyrimidine (PP2) was purchased from Calbiochem. The anti-CD3ε mAb (clone145-2C11) and the CTLA4–Fc fusion protein were supplied by Becton Dickinson. The antibodies used for FACS analysis were as follows: anti-CD28-PE (37.51) and anti-CD80-PE (16-10A1) from eBioscience, anti-CD80-APC (16-10A1), anti-CD54-APC (YN1/1.7.4), anti-CD4-Pacific Blue (GK1.5), anti-CD25-APC (PC61) and anti-CD62L-Pecy7 (MEL-14) from BioLegend, anti-CD44-PE (IM7), anti-I-A^k^-PE (11-5.2) and anti-CD69-FITC (H1 2F3), anti-CD11a-PE (Clone 2D7) from BD Biosciences.

### Transfection of B7-1 and ICAM-1

pSelectBlastimB7.1 and pUNO1-mICAM1 plasmids (from Invivogen) encoding for mouse B7-1 and mouse ICAM-1, respectively, were used for transfecting (Amaxa, V solution, A024) the COS-A^k^ cells (16). Cells were sorted based on their staining with specific antibodies. The resulting stable transfectants were referred as COS-A^k^/B7-1 and COS-A^k^/ICAM-1, respectively. The surface density of B7-1 in COS-A^k^/B7-1 was estimated to be ~10 molecules/μm^2^ by using anti-CD80-PE and QuantiBRITE beads (BD Biosciences), which was within the range of that found on professional APCs *in vivo* (17).

### Flow Cytometry

Cells were analyzed on a BD LSR II flow cytometer using the FlowJo software. Cells sorting were performed on BD Aria III. To quantify the antigen molecules on cell surface, BD Quantibrite™ PE Beads (BD Biosciences) were used according to the manufacturer's manual.

### T Cell Isolation

CD4^+^ T cells were purified from the total cell population of pooled lymph nodes and spleens from 3A9 TCRtg mice using Dynabeads Untouched Mouse CD4 cell kits (Life Technologies), after cell extraction on nylon membrane and lysis of erythrocytes with NH_4_Cl. All cells were 3A9 TCR positive. The naive T cell (CD44^lo^CD62L^hi^CD25^−^CD69^−^) population was generally >95%.

### Measurement of IL-2 Production From T Cell Stimulated

Purified CD4^+^ T cells from 3A9 TCRtg mice were co-cultured at 37°C with COS APC cells, with different doses of HEL 48-63 peptides. T cells were harvested after 20 h. The concentration of IL-2 in the supernatants was measured by Mouse IL-2 ELISA Ready-SET- Kit (eBioscience).

### The Recording of the T Cells Stained With Calcium Indictor Dye

The measurement of CD4^+^ T cell intracellular calcium was performed using MAAACS (methods for automated and accurate analysis of cell signals) (15). In brief, T cells were first loaded with calcium indicator dye (BD™ PBX Calcium Assay Kit) at 37°C for 30 min protected from light. After washing twice in HBSS (Hank's buffer salt solution) containing 1 mM HEPES (pH 7.4), T cells resuspended in the same solution plus CaCl_2_ and MgCl_2_ were added onto the monolayer of COS APCs in Lab-Tek chamber slides (Nalge Nunc International). Time-lapse movies of the cells, composed of 420 images with each taken every 7 s, were performed on a Zeiss LSM 510 Meta confocal microscope equipped with a C-Apochromat 40X/1.2 water immersion objective as well as an argon laser with a 488 nm dichroic and a 505 nm long pass filter. During the movie recording, cells were kept at 37°C.

### Analyses of the T Cell Movement

From recorded movies, T cell instantaneous speeds were calculated as previously described using MAAACS (15). The cell trajectories analyzed by MAAACS were also used to calculate the mean square displacement (MSD) values, which were computed using MSDAnalyzer freely available on GitHub, a Matlab (The MathWorks, Natick, MA) package accordingly to Tarantino et al. (18). The log-log MSD curves were fitted with a linear function to retrieve the so-called α value reflecting the mean cell motion behavior. α close to 1 means a normal diffusive movement, while a value < 1 indicates a subdiffusive movement and values > 1 a superdiffusive movement of the cells.

### The Analysis of the T Cell Calcium Response Magnitude and Types

Experimental raw fluorescence images were first median-filtered and then correlated with a cylindrical mask, transforming pseudo round objects into Gaussian peaks while retaining the overall initial intensity. Note that the mask width is set to match the average radius of the cell. The trajectories are rebuilt by connecting frame to frame cell positions using the MTT algorithm (19). The construction of the raw fluorescence intensity distribution histogram prior to the maximal amplitude allowed to compute a median that is defined as the individual baseline value to which raw intensities then are normalized. The mean amplitude (MA) of the fluorescence intensities corresponds to the average normalized intensity over the whole trace. From the average MA of the cell population, an activation threshold could be established by comparing the stimulating and non-stimulating conditions. This was determined as a combination of maximizing the probability of detection (PD), and minimizing the probability of false alarm (PFA). Based on our previous work using MAAACS to study the calcium responses in 3A9 TCRtg CD4^+^ T cells (15), we set this threshold to 2, and verified that when preforming analyses with this threshold, while the PD was very high (>0.96), the PFA was kept low (<0.04) for reacting cells under all stimulation conditions (data not shown). A number of parameters were then established for the calcium response. The activated (or reacting) cells are cells whose normalized fluorescent intensity spikes reached the threshold value in a given condition. The magnitude of calcium response for each reacting cell was quantified using two parameters, i.e., MA as already mentioned, and the response fraction (RF), corresponding to the ratio between the time when the intensity is above the threshold and the total time during which the intensity is detected. It could be noted that MA may be <2, because MA is the mean normalized intensity value of a recording. Finally, the calcium responses displayed by the active cells were classified into three types according to the level of the RF (15). The “maintained” type was defined as RF higher than 0.8, while the “unique” type was defined as RF lower than 0.2 with a single burst. In other cases, the type is “oscillatory.”

### The Autocorrelation Analysis of the Calcium Signal Amplitudes

The autocorrelation analysis was performed with the following steps:

a: *data detrending*
  In our analysis, the lagged difference was used as the data detrending method to ensure that the detrended time series were first-order and second-order stationary after removing the trend of the raw data (*y*_*t*_) along with time. We obtained the stationary process *X*_*t*_ by the first (or higher) order differences, for instance, for the first order differences calculation:
  $$\begin{array}{l} X_{t}=y_{t}-y_{t-1} \end{array}$$
  And higher order difference is needed until the stationary condition is satisfied.
b: *Autoregressive model*
  Each time series was then decomposed into the deterministic and purely stochastic parts by fitting an autoregressive (AR) model with the following form:
  (1) $$\begin{array}{r} X_{t}=\sum_{l}^{P}\alpha_{l}X_{t-l}+\varepsilon_{t} \end{array}$$
  where parameter α_*p*_ represents the correlation between *X*_*t*_ and *X*_*t*−*p*_, and the error term ε_*t*_ refers to the disturbance with mean of zero. The optimal lag order p is chosen according the Schwartz Bayesian Criterion (SBC). We obtained the correlation coefficients via Yuler-Walker method (which is invulnerable to non-normality of the error term but requires the zero mean) and then use them to analyze the typical oscillation and decay times of signals. The Equation (1) could be rewritten into a compact form so that:
  (2) $$\begin{array}{r} {\tilde{X}}_{t}=Ã{\tilde{X}}_{t-l}+{\tilde{\epsilon }}_{t} \end{array}$$
  Where:
  (3) $${\tilde{X}}_{t}=\left(\begin{array}{c} X_{t}\\ X_{t-1}\\ \vdots \\ X_{t-p+1} \end{array}\right)\in {\mathbb{R}}^{p}and\;{\tilde{\epsilon }}_{t}=\begin{array}{c} \;\left(\begin{array}{c} \varepsilon_{t}\\ 0\\ \vdots \\ 0 \end{array}\right) \end{array}\in {\mathbb{R}}^{p}$$
c: *Construction of the* Ã *matrix* (20)
  (4) $$With\;\tilde{A}=\left[\begin{array}{ccccc} \alpha_{1} & \alpha_{2} & \cdots & \alpha_{p-1} & \alpha_{p}\\ 1 & 0 & \cdots & 0 & 0\\ 0 & 1 & \cdots & 1 & 0\\ 0 & 0 & \ddots & 0 & 0\\ 0 & 0 & \cdots & 1 & 0 \end{array}\right]\in {\mathbb{R}}^{p^{*}p}$$
  The dynamics of the coefficients ${\tilde{X}}_{t}^{\left(p\right)}$ are then characterized by the combination of *p* univariate AR (1) models given as:
  $$\begin{array}{l} {\tilde{X}}_{t}^{\left(l\right)}=\lambda_{l}{\tilde{X}}_{t-1}^{\left(l\right)}+{\tilde{\varepsilon }}_{t}^{\left(l\right)},\;l=1,\ldots ,p \end{array}$$
  By taking the temporal average, we obtain the dynamic of the expanded values of the coefficients:
  $$\begin{array}{l} \langle {\tilde{X}}_{t}^{\left(l\right)}\rangle =\lambda_{l}\;\langle {\tilde{X}}_{t-1}^{\left(l\right)}\rangle \end{array}$$
  and which describes a spiral
  $$\begin{array}{l} \langle {\tilde{X}}_{t}^{\left(l\right)}\rangle =\lambda_{l}^{t}\;\langle {\tilde{X}}_{t-1}^{\left(l\right)}\rangle =e^{-t/\tau_{l}}e^{\left(\arg\lambda_{l}\right)it}\;\langle {\tilde{X}}_{t-1}^{\left(l\right)}\rangle \end{array}$$
d: *Decomposition*
  Thus, we could decompose the coefficient matrix Ã
  $$\begin{array}{l} Ã=\tilde{S}\Lambda {\tilde{S}}^{-1} \end{array}$$
  where $\tilde{S}$ is a non-singular matrix with the columns of which correspond to the eigenvectors and Λ is the associated diagonale matrix whose diagonal is composed by the eigenvalues λ_*l*_
e: *Decaying*
  Decaying is a function of λ_*l*_ and the maximum value of decay time is what we need
  With decay times:
  $$\begin{array}{l} \tau_{l}=-1/\log|\lambda_{l}| \end{array}$$

### Statistical Analysis

All statistical analyses were performed using GraphPad Prism 7.0. A non-parametric two-tailed unpaired Mann–Whitney test was used for comparison between two groups.

## Results

### Concomitant Stimulation of the TCR and CD28 With Their Cognate Ligands Expressed on COS-7 Cells Trigger an Efficient Activation of Naive CD4^+^ T Cells

To analyze the TCR and CD28 interactions with their respective ligands, we used naive CD4^+^ T cells from 3A9 TCRtg mice (14), and COS-7 fibroblasts (derived from monkey kidney tissue) stably transfected with mouse MHC class II (MHCII) I-A^k^ (designated as COS-A^k^) alone or together with mouse B7-1 (COS-A^k^/B7-1) as the antigen-presenting cells (APCs). 3A9 TCR is specific for the hen egg lysozyme (HEL) peptides, such as HEL48-63, presented by I-A^k^. We have previously shown that the I-A^k^-expressing COS-A^k^ cells enabled efficient presentation of HEL peptides to T cell hybridoma cells expressing the 3A9 TCR (15). Moreover, given the fact that COS-7 cells are known to be devoid of ascribed ligands for T cell costimulatory or adhesion receptors (21, 22, 23), or T cell costimulation promoting activity (24), COS-A^k^/B7-1 cells allowed us to analyze the contribution of CD28-B7 interactions in T cell activation. In addition, we generated COS-A^k^/ICAM-1 in order to compare the effects of B7-1 and ICAM-1 in T cell activation as ICAM-1 binding to LFA-1 was known to facilitate TCR–pMHC interactions and strengthen TCR-triggered signaling pathways. The different cell lines were sorted according to their surface expression of I-A^k^, B7-1, and ICAM-1. As a result (Figure S1), all the cell lines expressed the same level of I-A^k^ on their surface. In addition, the relative surface expression of B7-1 on COS-A^k^/B7-1 and ICAM-1on COS-A^k^/ICAM-1 were comparable. On the other hand, and as expected, mouse naïve CD4 T cells had a much higher surface level of LFA-1 than CD28 (Figure S1).

**Figure 1:**
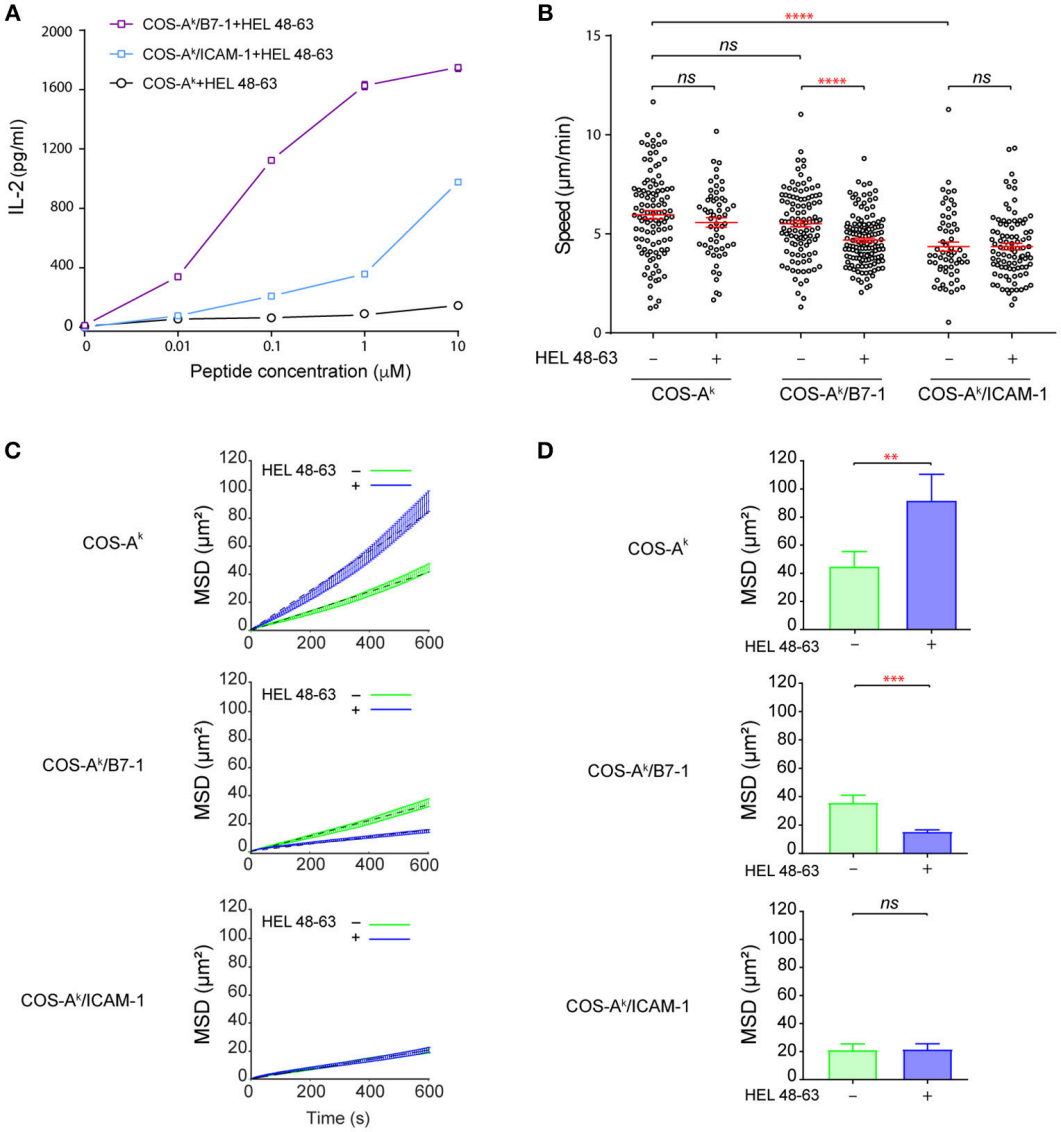
B7-1 expression on COS APCs provides efficient costimulatory signaling to naive CD4^+^ T cells. **(A)** Activation of naive CD4^+^ T cells upon contact with COS APCs loaded with HEL 48–63 peptides. 5 × 10^5^ naive CD4^+^ T cells from 3A9 TCRtg mice were cultured at 37°C with COS-A^k^, COS-A^k^/B7-1, COS-A^k^/ICAM-1 cells, loaded or not with different doses of HEL 48–63. T cells were harvested after 20 h. IL-2 concentration in the supernatants was measured by ELISA. Data represented the mean of three individual experiments. Data: mean ± s.d. **(B)** Naive CD4^+^ T cells form mobile contacts with COS APCs loaded or not with HEL 48–63. Naive CD4^+^ T cells from 3A9 TCRtg mice were loaded onto APCs monolayers of COS-A^k^, COS-A^k^/B7-1, COS-A^k^/ICAM-1 cells grown to confluency on Lab-Tek chamber slide, loaded or not with 1 μM HEL 48–63. T cell trajectories were recorded for 45 min at 37°C using confocal video-microscope. Average migration speed over the whole trajectory was calculated as described in Materials and Methods. Data: mean ± SEM. Their numerical values were 5.96 ± 0.21 μm/min (*n* = 105), 5.57 ± 0.24 μm/min (*n* = 56), 5.59 ± 0.18 μm/min (*n* = 111), 4.68 ± 0.11 μm/min (*n* = 134), 4.36 ± 0.23 μm/min (*n* = 60), and 4.36 ± 0.17 μm/min (*n* = 93) for the T cells contacting with COS-A^k^, COS-A^k^ plus HEL 48–63, COS-A^k^/B7-1, and COS-A^k^/B7-1 plus HEL 48–63, COS-A^k^/ICAM-1, and COS-A^k^/ICAM-1 plus HEL 48–63, respectively. (**C)** Mean square displacement over time and **(D)** average mean square displacement over 10 min of T cells seeded onto COS-A^k^, COS-A^k^/B7-1, and COS-A^k^/ICAM-1 cells, loaded or not with 1 μM HEL 48–63, respectively. Mann–Whitney tests were performed using Graphpad prism 7.0 for comparisons between two groups. Statistical significance was set at 0.05, and *p*-values < 0.05 were denoted in red. **p < 0.01, ***p < 0.001, and ****p < 0.0001.

The incubation with COS-A^k^ cells loaded with HEL peptides resulted in inefficient activation of naive 3A9 TCRtg CD4^+^ T cells, as measured by IL-2 secretion. However, the incubation with COS-A^k^/B7-1 cells loaded with HEL peptides to concurrently trigger TCR and CD28 signaling pathways elicited strong activation of 3A9 TCRtg CD4^+^ T cells (Figure 1A). In addition, we observed that COS-A^k^/ICAM-1 cells also could support the activation of 3A9 TCRtg CD4^+^ T cells when pulsed with HEL peptides, even if the strength of activation appeared to be less strong than with COS-A^k^/B7-1 cells. Interestingly, video microscopic examination revealed that 3A9 TCRtg CD4^+^ T cells patrolled quickly on the surface of the COS APCs with or without antigenic peptides, forming transient, and mobile contacts (Figure 1B and Figure S2A) with the average instantaneous speed values that were higher than 4.0 μm/min. Such mobile and short-lasting contacts between T cell and APCs where the TCR binds to pMHC have been referred as immunological kinapses (25) [in which the average T cell speed > 2.5 μm/min (26)], differentiating them from immunological synapses, which are long-lived and stable [average T cell speed < 2.5 μm/min (26)]. Various studies have shown that for naive T cells, immunological kinapses are probably the most frequent type observed *in vivo* (26–30) and *in vitro* (26), at least during the early phase of T cell activation. Our analysis also showed that the average speed of 3A9 TCRtg CD4^+^ T cells was the same with COS-A^k^ cells loaded or not with HEL peptides; a slight reduction was found with COS-A^k^/B7-1 cells after HEL peptide loading, and the lowest speed was observed with 3A9 TCRtg CD4^+^ T cells interacting with COS-A^k^/ICAM-1 cells, either before or after peptide loading (Figure 1B). We next conducted the mean square displacement (MSD) vs time analyses (31) of the CD4 T cell movements on different COS APCs between non-stimulating and stimulating conditions (Figures 1C,D and Figure S2B). Of interests, these analyses revealed that the antigen stimulation affected the MSD of CD4 T cells that interacted with COS-A^k^ and COS-A^k^/B7-1 cells, respectively, but in an opposite direction (Figure 1D). Moreover, while these movements all contained heterogeneous diffusion modalities, they were all also largely dominated by the constrained (subdiffusive) type. However, an increase in the percentage of trajectories with an α value significantly > 1 suggested that a directed-like motion (superdiffusive) type was present in the movement of T cells upon stimulation by antigen in the absence of CD28 or LFA-1 signaling (Figure S2B). Finally, the free-like (normal diffusive) motion was essentially absent in all cases (Figure S2B).

### TCR Stimulation With pMHC Raises the Fluctuating Calcium Mobilization Level in Naive CD4^+^ T Cells

**Figure 2:**
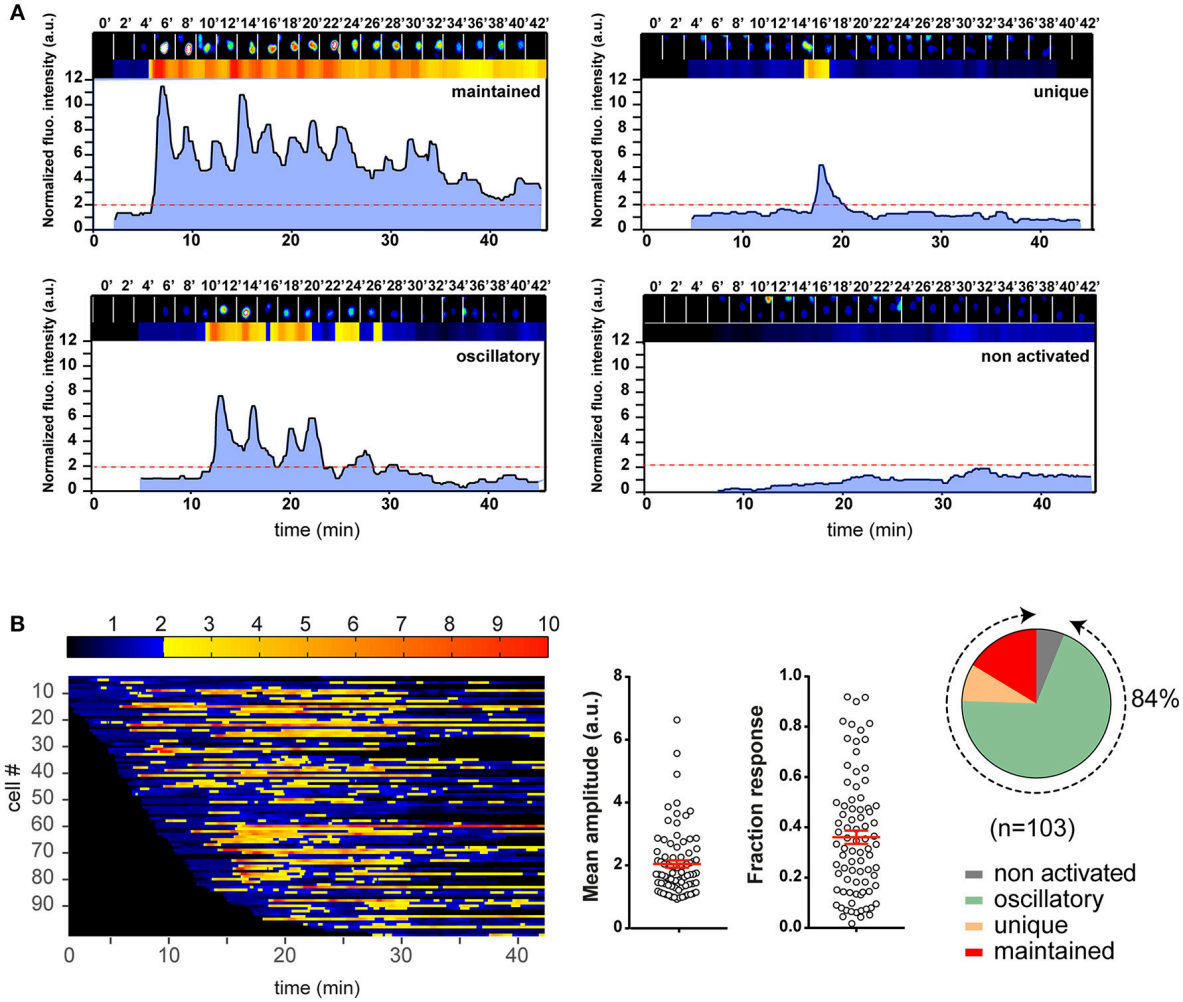
Illustration of the T cell calcium response analysis with an example experiment. Naive 3A9 TCRtg CD4^+^ T cells were loaded with Fluo-4 PBX before incubation at 37°C for 45 min with COS-A^k^ pulsed with 10 μM HEL 48–63. Time-lapse movies of T cells were made using confocal microscope as described in Materials and Methods. **(A)** Single cell fluorescence recordings analyzed by MAAACS and categorized into different classes according to the magnitude and shape of fluorescence signals. The examples of four different response types are shown. In each panel, the top row is made up of 2 min-delayed snapshots of raw images of a cell. Just below are the normalized fluorescence intensities displayed in the form of a bar code and a line profile, respectively. The non-activated cells are defined as cells whose normalized fluorescence intensities have never reached the activation threshold (set at 2.0, red dotted line, see Methods and Materials for more details) along the whole trace. For the activated cells, we defined a response as “maintained” when the response fraction (RF) was higher than 0.8, and “unique” when the response fraction was lower than 0.2 with a single burst. In all other situations, calcium responses were “oscillatory.” **(B)** Color barcoding and calculation of the analytical parameters of the calcium response. The normalized fluorescence amplitude of each cell is plotted along a horizontal line as a function of time with a color-coded intensity (dark to blue below the threshold of activation and yellow to red above the threshold of activation). The analytical parameters of the calcium response, i.e., the mean amplitude (MA) and RF calculated by MAAACS are plotted (mean ± SEM). The global overview of the cell response heterogeneity is summarized in the form of a pie chart.

**Figure 3:**
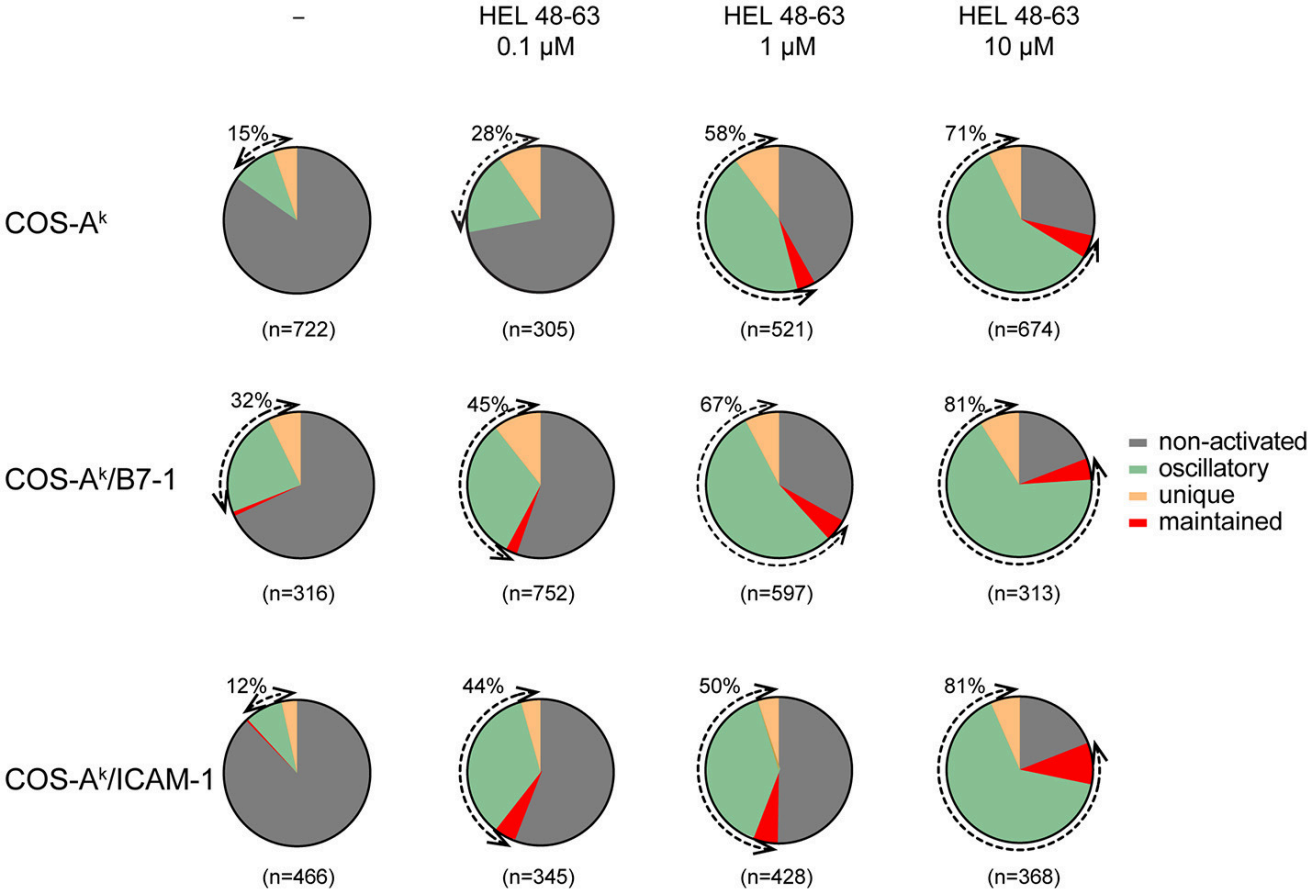
Naive CD4^+^ T cells show various calcium response modes upon different stimulation. Naive 3A9 TCRtg CD4^+^ T cells were loaded with Fluo-4 PBX before incubation at 37°C for 45 min with COS APC cells pulsed without or with different doses of HEL 48–63 as indicated. Time-lapse movies of T cells were made by confocal microscope as described in Materials and Methods. For each antigenic peptide concentration, modes of calcium response are represented as a pie chart: non-activated in gray, maintained in red, oscillatory in green, and unique in yellow. The total number of cells analyzed is indicated in parentheses. The percentage of activated (reacting) cells is shown by the double arrows, and their values are indicated.

**Figure 4:**
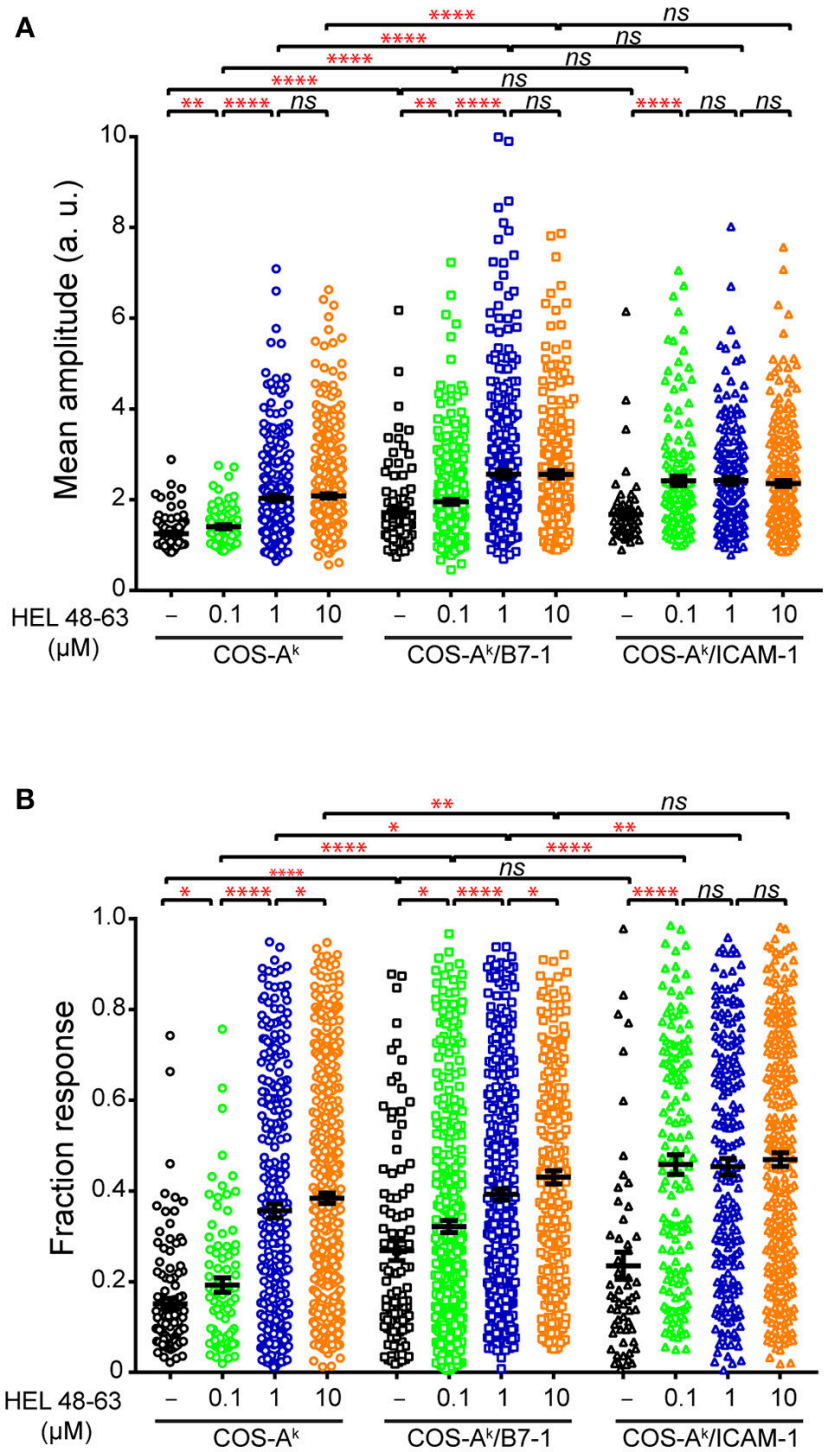
Comparisons of analytical parameters of the calcium response in naive CD4^+^ T cells upon different stimulation. Naive 3A9 TCRtg CD4^+^ T cells stained with Fluo-4 PBX were loaded onto COS APC monolayers at 37°C and observed for 45 min. COS-A^k^, COS-A^k^/B7-1, COS-A^k^/ICAM-1 APCs were loaded or not with 0.1, 1, and 10 μM HEL 48–63. Average mean amplitude **(A)** and responding fraction **(B)** were calculated as described in Materials and Methods. Data: mean ± SEM. Mann–Whitney tests were performed using Graphpad prism 7.0 for comparisons between two groups. Statistical significance was set at 0.05, and *p*-values < 0.05 were denoted in red. *p < 0.05, **p < 0.01, and ****p < 0.0001.

The calcium response is a major signaling event downstream to receptor-proximal signaling pathways in T cell activation (32, 33). Previous works have established that the TCR early signaling enables elevation of the intracellular calcium concentration ([Ca^2+^]_i_) that could be further potentiated upon CD28 coengagement (4, 10). Moreover, the results from several studies have suggested that enhanced calcium response plays an essential role in the costimulatory function of CD28 in T cell activation (2, 4, 10, 34). However, its involvement in the TCR- and CD28-mediated stimulation currently remains poorly known for the naive T cell activation. One important reason is that study models were built up either by inferring from observations made in other T cell types, or experimental data by using antibodies against the TCR and CD28, which may mimic some properties but certainly miss others of the corresponding natural ligands. We therefore decided to examine the calcium responses of 3A9 TCRtg CD4^+^ T cells upon interactions with COS APCs. For this, we used the MAAACS approach (15), an inclusive method that we previously developed, which enabled comprehensive analyses of the calcium dynamics in T cells that are constantly moving and forming kinapses with APCs. The Figure 2 and Figure S3 depict how the use of MAAACS to analyze the calcium mobilization in 3A9 TCRtg CD4^+^ T cells allowed the efficient determination of its frequency and average response magnitude that was determined by the average mean amplitude (MA) and response fraction (RF). Consistent with the previous study (15), we observed that when seeded onto COS-A^k^ cells, some 3A9 TCRtg CD4^+^cells exhibited weak and transient calcium elevation over a period of several tens of minutes (Figure 3). A factor that could account for such basal fluctuating [Ca^2+^]_i_ rises is T cells interacting with APCs, either with or without the involvement of MHC molecules (35–37). When 3A9 TCRtg CD4^+^ T cells were seeded onto COS-A^k^ cells loaded with HEL peptides, 3A9 TCRtg CD4^+^ T cells continued to experience fluctuating [Ca^2+^]_i_ elevations, in line with previous studies on naive CD4^+^ T cells upon TCR stimulation (15, 36, 38); however, there was an increase in both percentage and average response magnitude, as estimated by the MA and RF of the reacting cells, respectively (Figures 3, 4). In addition, we observed that these increases took place in a dose-dependent manner when peptides were loaded at concentrations of 0.1, 1.0, and 10 μM, respectively. Finally, for all the stimuli conditions, the main type of calcium response for activated cells continued to be “oscillatory,” the proportion of which increased with the peptide concentration (Figure 3).

### TCR and CD28 Concomitant Stimulation Induces a Stronger Calcium Mobilization Than Single-Receptor Stimulation in Naive CD4^+^ T Cells

We next examined the calcium mobilization of 3A9 TCRtg CD4^+^ T cells activated by concurrent TCR and CD28 stimulation. When 3A9 TCRtg CD4^+^ T cells were seeded onto COS-A^k^/B7-1 cells without HEL peptides, a weak but detectable increase in calcium elevations, in terms of both frequency and average response magnitude of the reacting cells, was observed compared with 3A9 TCRtg CD4^+^ T cells seeded onto COS-A^k^ cells, indicating that the engagement alone of CD28 enabled a low level of calcium mobilization (Figures 3, 4). When 3A9 TCRtg CD4^+^ T cells were incubated with COS-A^k^/B7-1 cells loaded with HEL peptides, with both TCR and CD28 being engaged, the calcium mobilization level was higher than that obtained with each receptor alone, as expected. In addition, the type of response with regards to the presence of antigen stimulus was consistent with that expected—that is, the response increased with the increased concentration of the HEL peptide, and the response attained its maximum level at the concentration of 10 μM. However, we noticed that the observed augmentation in the percentage and average mean response magnitude of reacting cells did not characterize a functional aspect that could account for the high costimulatory activity of CD28. Indeed, the costimulation of CD28 together with TCR stimulation triggered by a low concentration of HEL peptide (0.1 μM) resulted in a weaker calcium response in 3A9 TCRtg CD4^+^ cells than that observed with TCR stimulation alone triggered by a higher concentration of HEL peptide (10 μM); however, a much higher secretion rate of IL-2, was found in the first case than in the second (Figures 1A, 4).

Our following comparison between CD28 and LFA-1 in their cooperation with the TCR to elicit the calcium response further supported the above remark. We observed that the engagement of LFA-1 by its ligand ICAM-1 alongside the stimulation of TCR by pMHC resulted in a significant increase in calcium mobilization, whereby both frequency and average response magnitude of reacting cells reached plateau level with 0.1 μM peptides. Comparatively, co-triggering LFA-1 along with TCR stimulation produced a stronger calcium response in 3A9 TCRtg CD4^+^ cells than did CD28 (Figures 3, 4). However, co-engagement of CD28 caused a greater TCR-induced IL-2 secretion than the LFA-1. Indeed, co-engaging CD28 with the TCR that was stimulated with HEL peptides at 0.1 μM resulted in a higher IL-2 production than co-engaging LFA-1 with TCR stimulation with HEL peptides at any concentration (Figure 1A), despite the more potent calcium response magnitude occurred in the cases of LFA-1 co-engagement (Figure 4). Altogether, our data showed that both CD28 and LFA-1 stimulation could significantly potentiate the calcium response induced by the TCR–pMHC interactions. However, the increase of the frequency and average response magnitude in the calcium mobilization is probably not the most crucial facet of CD28 costimulatory function in T cell activation.

### TCR and CD28 Concomitant Stimulation Triggers a Distinctive Increase in the Decay Time (τ) of [Ca^2+^]_i_ Elevations as Shown by Time Series Analysis

**Figure 5:**
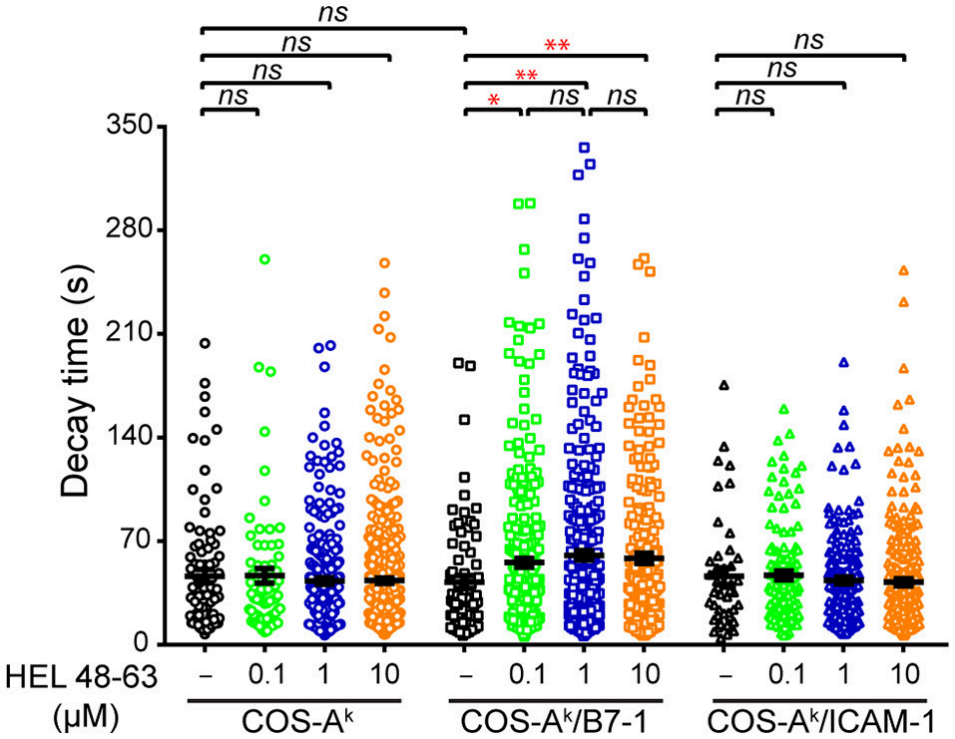
Comparisons of decay time (τ) of calcium response in naive CD4^+^ T cells upon different stimulation. Naive 3A9 TCRtg CD4^+^ T cells stained with Fluo-4 PBX were loaded onto COS APC monolayers at 37°C and observed for 45 min. APC cells were loaded or not with 0.1, 1, and 10 μM HEL 48–63. Analysis of average decay time (τ) was performed as described in Materials and Methods. Data: mean ± SEM. Mann–Whitney tests were performed using Graphpad prism 7.0 for comparisons between two groups. Statistical significance was set at 0.05, and *p*-values < 0.05 were denoted in red. *p < 0.05 and **p < 0.01.

Our observations made above could reflect that CD28 functions through mechanisms that are partly dependent on calcium mobilization or, alternatively, that CD28 costimulation crucially relies calcium mobilization, but in addition to the classical aspects—namely the reacting cell frequency and average response magnitude—there could be other molecular features. Given the results of previous studies (2, 4, 34), we assumed the latter to be the most likely possibility. We considered that CD28 costimulation could change the temporal dynamics of the calcium response induced with TCR stimulation alone (10, 34, 39). For instance, earlier investigations with human primary T cells using antibodies showed that concurrent TCR and CD28 ligation elicited a more-sustained calcium response than did each receptor individually (34, 39). However, such antibody-elicited T cell responses were not fluctuating, and their maintenance was generally characterized by a flatter slope in the signal decay, which differed from the calcium response found here. We therefore decided to conduct the autocorrelation analysis to reveal the dynamics of intracellular calcium mobilization in 3A9 TCRtg CD4^+^ T cells. The autocorrelation analysis examined the self-similarity of a time series signal (Figure S4), which can be characterized with a decay time (τ) that describes the persistence of the information that the signal carries. Such an approach was previously employed to examine the contribution of [Ca^2+^]_i_ oscillation during IFN-γ production by human cytotoxic T cell clones (40). Our autocorrelation analysis interestingly revealed that the calcium amplitude signal in the (reacting) 3A9 TCRtg CD4^+^ T cells at the basal conditions had an average characteristic decay time (τ) of ~46 s (Figure 5). The calcium signal decay time (τ) remained unchanged in 3A9 TCRtg CD4^+^ T cells upon stimulation of TCR, CD28, or LFA-1, with the corresponding ligands. However, stimulation of both TCR and CD28, but not both TCR and LFA-1, induced a striking increase of decay time (τ) to ~58 s (Figure 5). Moreover, the increase immediately reached a plateau value with 0.1 μM HEL peptide and kept unchanged at higher peptide concentrations, suggesting that the induction involves a very efficient mechanism that allows it to take place even when the level of antigen is low. Therefore, our analysis uncovered the augmented decay time (τ), representing a unique feature of the calcium response elicited by TCR and CD28 concurrent stimulation, and we suggest that the CD28 costimulatory activity thus contributes to producing a type of fluctuating intracellular calcium mobilization with greater incidence, higher magnitude, and longer memory of information it carries than TCR stimulation alone during naive T cell activation.

## Discussion

In this work, we investigated the mechanism of activation of naive CD4^+^ T cells mediated by the TCR and CD28. The T cell activation process has been the focus of numerous studies during past decades, but its mechanism is still only partially understood. Our current study has identified an important step in the TCR and CD28 signaling pathways where the two pathways interact and complement one another to promote the effective initiation of naive T cell activation—namely the regulation of the fluctuating intracellular calcium mobilization. Indeed, we have shown for the first time that simultaneous TCR and CD28 stimulation, but not stimulation of individual receptors, significantly extended the decay time (τ) of the calcium amplitude signal detected prior stimulation. This special property, uniquely characterizing the costimulatory action of CD28, probably contributes to TCR- and CD28-mediated signaling pathways leading to efficient T cell activation. This finding supports the notion that it is only after initial antigen recognition by TCR that CD28 efficiently triggers signaling pathways in naive T cells, given the very weak basal interactions of CD28 with B7 molecules (7), and its low abundance in this cell population (2, 3).

CD28 is a major costimulatory receptor that is constitutively expressed on naive T cells and is essential for the activation of naive T cells by antigen recognition. However, much information on the molecular mechanism of CD28 signaling in naive T cells is largely inferred from results obtained with T cell lines or antigen-experienced T cells but very rarely directly studied. This is mainly due to the fact that it remains technically more challenging to study early signaling events following stimulation of surface receptors in naive T cells. However, it is not clear to what extent the information obtained from these studies can be applied directly to the activation of naive T cells, as the early signaling steps induced upon T cell antigen recognition between naive T cells and stimulation-experienced T cells exhibit both qualitative and quantitative differences. In addition, there is also the problem that, in many of these studies, receptor stimulation has been achieved only by means of antibodies instead of physiological ligands. It is reasonable to consider that antibodies, especially in soluble form, can only mimic some, but not all, of the key actions of membrane-bound ligands (41, 42). It is therefore important to examine the mechanism of activation of naive T cells via TCR and CD28 by directly using naive cells that are triggered by natural ligands on APCs, which we have done here.

Calcium mobilization is known to be an essential signaling event in T cell activation (32, 33). Previous studies have suggested that the contribution of CD28 in calcium mobilization is essential to its co-stimulatory activity in promoting T cell activation. Naive T cells exhibit significant basal intracellular Ca^2+^ transients both *in vitro* and *in vivo* (35–37). The considerable fluctuation of [Ca^2+^]_i_ rises also feature the calcium responses in naive T cells when exposed to various types of external stimuli (15, 38, 36). We utilized in our study MAAACS, an inclusive method that we recently developed that allows efficient and robust analysis of the calcium responses in naive mouse CD4^+^ T cells. We observed that in the activation of naive T cells, the concurrent engagement of the TCR and CD28 with their cognate ligands elicited an elevation in the fluctuating [Ca^2+^]_i_ with both higher frequency and magnitude than did TCR or CD28 engagement alone, which was not surprising. However, by examining the autocorrelation function of the calcium amplitude signal (40), we uncovered for the first time that there was an increase in the decay time (τ) of such signal from the basal level specifically after concurrent TCR and CD28 stimulation. The autocorrelation function normally describes the correlation between the signal observed at one time point with the observed at several previous time points and the decay time (τ) derived from this analysis characterizes the time length within which the signals at two time points show correlation. The decay time (τ) therefore provides information on the existence of a memory for the signal and its decay rate. It was found that the fluctuating calcium rises in mouse naive CD4^+^ T cells show an average decay time of ~46 s under basal conditions, which remained unchanged when the same cells were stimulated via either TCR, CD28, LFA-1, or TCR plus LFA-1 by the corresponding ligands, respectively. The combined stimulation by TCR plus CD28, however, resulted in a significantly longer decay time of ~58 s. This unique capacity from the concurrent TCR and CD28 stimulation may be linked to the key co-stimulatory molecule statue of CD28 critical to TCR-induced naive T cell activation upon pMHC engagement.

The origin of the prolonged decay time currently is unknown. One possible cause is because of the existence of constrained diffusions of intracellular Ca^2+^ ions due to spatial confinements for local and specific activation processes (43, 44). This could be arisen from generation of specific calcium nano- or microdomains at the vicinity of Ca^2+^ channels in membranes (45–47), or calcium flux in subcellular organelles such as nuclei (43, 48) and mitochondria (46, 49), etc. Interestingly, our present work is reminiscent for several aspects to the previous characterization of the role of CD28 co-stimulatory signaling in calcium responses by the previously activated mouse CD4^+^ T cells. In particular, Andres et al. (10) have found that CD28 co-stimulation upon binding to B7 strongly increased both the frequency and magnitude of TCR-induced calcium response upon binding to physiological levels of antigens during the T cell activation. In absence of CD28 co-stimulation, the predominant pattern of calcium influx triggered by the TCR was both attenuated in amplitude and transient in duration. It would therefore be interesting to examine whether the observed more persistent calcium response pattern associated with a productive activation signal in general displays an augmented decay time.

It was interesting to observe that, in our study, unlike the co-engagement of CD28 by B7-1, co-engagement of LFA-1 by ICAM-1 along with TCR engagement by pMHC did not extend the decay time (τ) of calcium amplitude signal as compared to the basal level in naive CD4^+^ cells. This occurred despite the co-engagement of LFA-1 with TCR engagement produced a stronger calcium response, both in terms of the frequency and magnitude than the co-engagement of CD28 (Figures 3, 4). Future studies will be needed to see if this remarkable functional difference between CD28 and LFA-1 also applies to other T cell populations. In this context, it noteworthy that a previous work has shown that in mouse naive CD8^+^ cells, that LFA-1 and CD28 exhibit distinct, non-overlapping ways to influence T cell activation (50).

Our data suggest that the interplays between the TCR and CD28 early signaling generate the coincidence detection mechanism for the initiation of naive T cell activation. This endows naive T cells with the ability to tightly control their activation, which is just as important as the ability to trigger activation. For instance, B7 molecules are expressed on dendritic cells and macrophages in lymphoid tissue, the levels of which are upregulated during infection and inflammation (17); their inappropriate binding to and stimulation of CD28 could have adverse pathological consequences. On the other hand, interplays at the early signaling steps could facilitate the TCR and CD28 to trigger balanced signaling pathways and their integration. At the cell population level, coincidence detection would be a mechanism that allows T cells to focus rapidly and efficiently on APC cells that have captured antigens and upregulated B7 and MHC molecules simultaneously, as in the case of dendritic cells stimulated with microbial products. Thus, our present work has provided strong support for a “privileged” costimulatory molecule role of CD28, the only B7 receptor constitutively expressed on naive T cells, in the activation of this T cell population.

## Author Contributions

X-JG, FX, C-RQ, and ZX designed the study, performed the experiments and analyzed the results. YH, A-MS, AF, SM, M-CP, CB, SJ, and JN assisted with realization and interpretation of the experiments, and provided several reagents. X-JG and H-TH supervised and directed the research. X-JG, FX, ZX, YH, SM, and H-TH wrote the manuscript. All authors discussed the results and commented on the manuscript.

### Conflict of Interest Statement

The authors declare that the research was conducted in the absence of any commercial or financial relationships that could be construed as a potential conflict of interest.
