# Supplementary material for: TCR and CD28 Concomitant Stimulation Elicits a Distinctive Calcium Response in Naive T Cells
Source: Front Immunol. 2018 Dec 4;9:2864. doi: 10.3389/fimmu.2018.02864 (PMC6288997; doi:10.3389/fimmu.2018.02864)
Supplement: Supplementary file 1 [file Data_Sheet_1.pdf]

Supplementary Information

TCR and CD28 concomitant stimulation elicits a distinctive calcium response in naive T cells

Fan Xia[1,6§], Cheng-Rui Qian[1,6&], Zhou Xun[2,3], Yannick Hamon[1], Anne-Marie Sartre[1], Anthony Formisano[1], Sébastien Mailfert[1], Marie-Claire Phelipot[1], Cyrille Billaudeau[1#], Sébastien Jaeger[1], Jacques A. Nunès[4,5], Xiao-Jun Guo[1,7*] and Hai-Tao He[1,7*]

[1]*Aix Marseille Univ, CNRS, INSERM, CIML, Marseille, France*

[2]*School of Economics, Jiangxi University of Finance and Economics, Nanchang, Jiangxi, China*

[3]*Aix Marseille Univ, AMSE and GREQAM, Marseille, France*

[4]*Aix Marseille Univ, CNRS, INSERM, Institut Paoli-Calmettes, CRCM, Marseille, France*

[5]*Centre de Recherche en Cancérologie de Marseille, Immunology and Cancer, Equipe Labellisée Fondation pour la Recherche Médicale, Marseille, France.*

[6]*These authors contributed equally to this work*

[7]*These authors contributed equally to this work*

***Correspondence:** Xiao-Jun Guo guo@ciml.univ-mrs.fr or Hai-Tao He he@ciml.univ-mrs.fr

[§]Present address: Department of Radiation Oncology, Fudan University Shanghai Cancer Center; Department of Oncology, Shanghai Medical College, Fudan University, Shanghai 200032, China

[&]Present address: Shanghai Blood Center, 1191 Hong Qiao Road, Changning District, Shanghai, China

[#]Present address: Micalis Institute, INRA, AgroParisTech, Université Paris-Saclay, 78350, Jouy-en-Josas, France

The Supplementary Information includes four supplementary figures (Supplementary Figures S1 – S4)

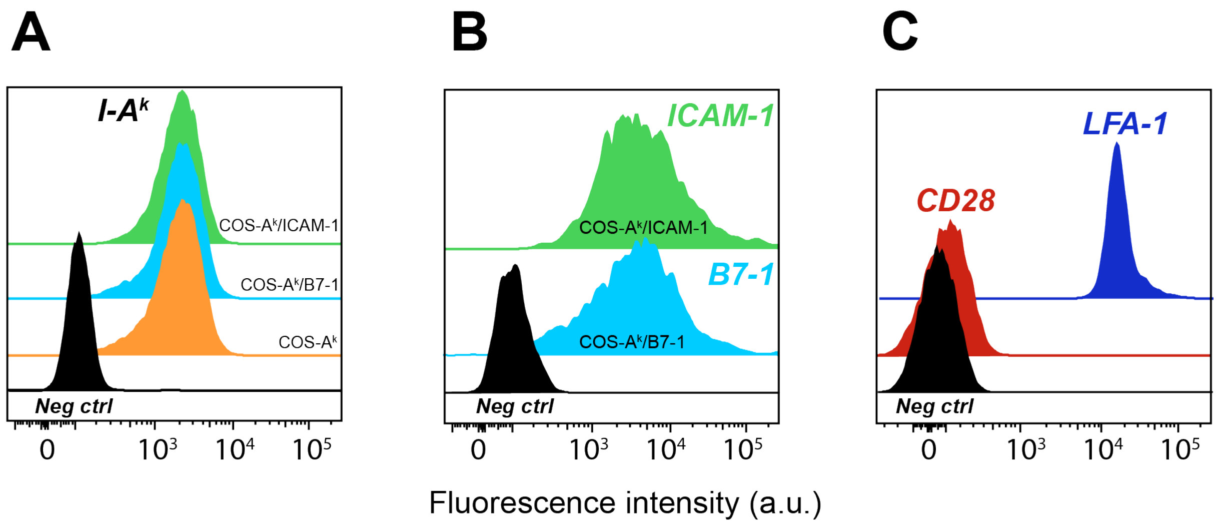

Figure S1. Flow cytometry analysis of COS APCs and 3A9 TCRtg CD4$^+$ T cells. (A) Expression of I-A^k on COS-A^k, COS-A^k/B7-1, COS-A^k/ICAM-1 cells, respectively. (**B**) Expression of B7-1 on COS-A^k/B7-1 and ICAM-1 on COS-A^k/ICAM-1 cells, respectively. (**C**) Expression of CD28 and LFA-1 on 3A9 TCRtg CD4$^+$ T cells. In these experiments, we used PE- (for I-A^k, CD28 and LFA-1) and APC- (for ICAM-1 and B7-1) conjugated mAbs that all display the protein/dye ratio at about 1:1 according to the suppliers' communications. In (**B**) and (**C**) we used the labeling mAbs at concentrations close to the saturating binding conditions in order to better compare the relative expression levels of different surface antigens. All data are representatives of three independent experiments.

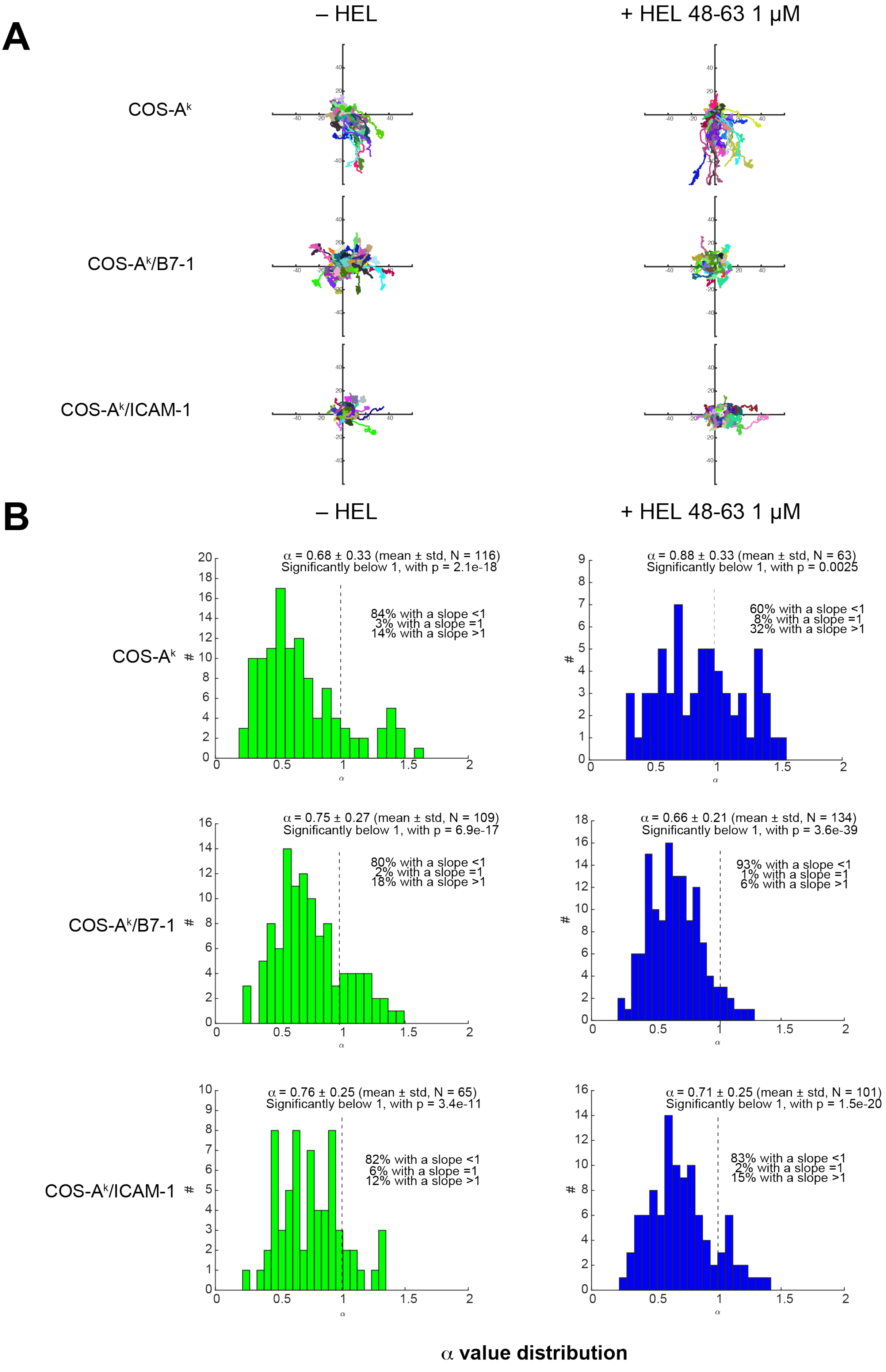

A
– HEL
+ HEL 48-63 1 µM
COS-A^k
COS-A^k/B7-1
COS-A^k/ICAM-1
B
– HEL
+ HEL 48-63 1 µM
α = 0.68 ± 0.33 (mean ± std, N = 116)
Significantly below 1, with p = 2.1e-18
84% with a slope <1
3% with a slope =1
14% with a slope >1
α = 0.88 ± 0.33 (mean ± std, N = 63)
Significantly below 1, with p = 0.0025
60% with a slope <1
8% with a slope =1
32% with a slope >1
α = 0.75 ± 0.27 (mean ± std, N = 109)
Significantly below 1, with p = 6.9e-17
80% with a slope <1
2% with a slope =1
18% with a slope >1
α = 0.66 ± 0.21 (mean ± std, N = 134)
Significantly below 1, with p = 3.6e-39
93% with a slope <1
1% with a slope =1
6% with a slope >1
α = 0.76 ± 0.25 (mean ± std, N = 65)
Significantly below 1, with p = 3.4e-11
82% with a slope <1
6% with a slope =1
12% with a slope >1
α = 0.71 ± 0.25 (mean ± std, N = 101)
Significantly below 1, with p = 1.5e-20
83% with a slope <1
2% with a slope =1
15% with a slope >1
α value distribution

Figure S2. T cell movements on the COS APCs. (A) Trajectories of naive $CD4^+$ T cells upon contacting with APCs loaded or not with HEL 48-63 peptides. Naive $CD4^+$ T cells from 3A9 TCRtg mice were loaded onto monolayers of COS-A^k, COS-A^k/B7-1, COS-A^k/ICAM-1 APCs grown to confluency on Lab-Tek chamber slides, loaded or not with 1 μM HEL 48-63. T cell trajectories were recorded for 45 min at 37 °C using a confocal video-microscope. **(B)** Distribution of α values from the MSD analyses.

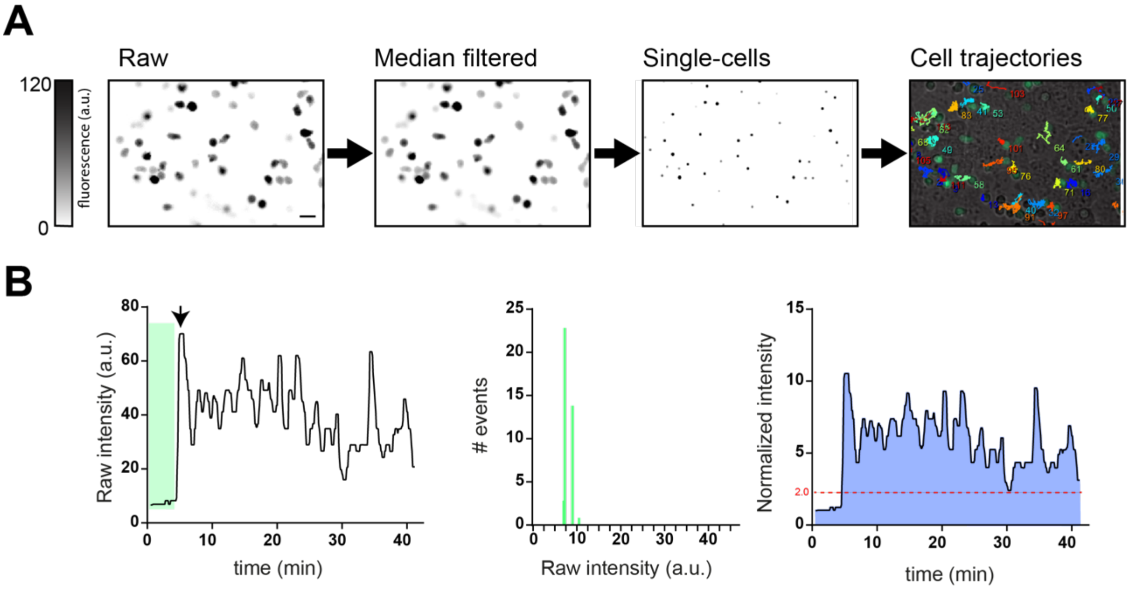

Figure S3. The mean amplitude (MA) and response fraction (RF) of the calcium response magnitude as determined with MAAACS. (A) Automatic tracking of fluorescent cells. Experimental raw fluorescence images (color coded with a white to black palette) were transformed into Gaussian intensity peaks as described in Materials and Methods. Scale bar, 10 µm. (**B**) MA and RF determination. The construction of the raw fluorescence intensity distribution histogram (green area in the left panel) prior to the maximal amplitude (arrow) allows to compute a median (6.69 in this example) that is defined as the individual baseline value to which raw intensities are normalized (right panel). The mean amplitude (MA) of fluorescence intensities corresponds to the average normalized intensity on the whole trace (blue area). Based on the analysis of the average MA of the cell population under stimulating and non-stimulating conditions, we could establish an activation threshold (red dotted line; in our study, this value was set 2, as described in Materials and Methods). The response fraction (RF) could then be determined as the ratio between the time when the intensity is above the threshold and the total time during which the intensity is detected. The experimental data were taken from the same experiment shown in Figure 2.

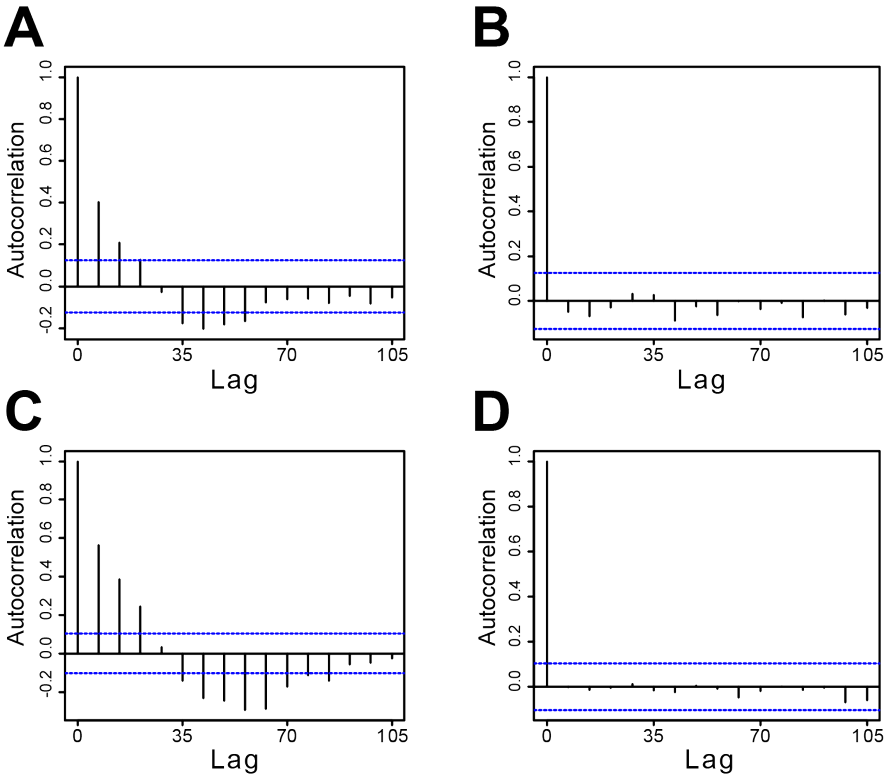

Figure S4. The examples of autocorrelation function analysis of the $[Ca^{2+}]_i$ time series data. 3A9 TCRtg $CD4^+$ T cells stained with Fluo-4 PBX were loaded respectively onto COS-A^k (**A** & **B**) and COS-A^k/B7-1(**C** & **D**), both pulsed with 1 μM HEL 48-63, and observed for 45 min at 37 °C. The autocorrelation function plots of $[Ca^{2+}]_i$ time series data are shown in (**A**) and (**C**), and the cumulative periodograms of the residual obtained by using AR fitting are shown in (**B**) and (**D**).
